# An objective measure for the assessment and management of fluid shifts in acute major burns

**DOI:** 10.1186/s41038-017-0105-9

**Published:** 2018-01-17

**Authors:** Pippa Kenworthy, Michael Phillips, Tiffany L. Grisbrook, William Gibson, Fiona M. Wood, Dale W. Edgar

**Affiliations:** 1 Fiona Wood Foundation, Fiona Stanley Hospital, Perth, Western Australia Australia; 2 School of Physiotherapy and Exercise Science, Curtin University, Perth, Western Australia Australia; 3 Burns Service of Western Australia, Fiona Stanley Hospital, Perth, Western Australia Australia; 4 Burn Injury Research Node, Notre Dame University, Fremantle, Western Australia Australia; 5 Harry Perkins Institute of Medical Research, The University of Western Australia, Perth, Western Australia Australia; 6 School of Physiotherapy, Notre Dame University, Fremantle, Western Australia Australia; 7 Adult State Burns Service, Fiona Stanley Hospital, Murdoch Drive, Murdoch, Western Australia 6150 Australia

**Keywords:** Bioelectrical impedance, Oedema, Wounds, Fluid resuscitation, Dressings

## Abstract

**Background:**

Major burns are life threatening. Fluid resuscitation is required for survival to maintain intravascular volumes and prevent hypovolemic shock. Bioimpedance spectroscopy (BIS) has been recognised as a potential method of monitoring fluid shifts after burn and in other disease states. The aims of this study were to examine the reliability of BIS across different dressing conditions and electrode positions, establish the influence of Acticoat™ on BIS variable measures and determine the validity of whole-body BIS to assess net fluid shift in the presence of moderate to major burns.

**Methods:**

An observational longitudinal cohort study was conducted from December 2014 to February 2016. Patients with over 15% total body surface area (TBSA) burns and injury less than 48 h were enrolled in the study. BIS triplicate measures were collected in an open wound and with an Acticoat^TM^ dressing (at 5 half hour intervals). Standard and alternate electrode placements were utilised for the reliability analysis and standard placement only for determining the validity of BIS in moderate to major burns. The ImpediMde SFB7 was used to collect whole-body and segmental BIS measures. Stata statistical software, release 14 was utilised to analyse all results. Descriptive analyses were performed and were reported using the means and standard deviations (SD).

**Results:**

BIS-repeated measures established BIS raw resistance (R), and predicted volume variables were reliable in any condition (intra-class correlation coefficient (ICC) 0.996–0.999, 95% confidence intervals (CI) 0.996–0.999) without a systematic difference. Acticoat™ dressings significantly influenced all BIS-predicted volumes (*p* ≤ 0.01) as determined by multilevel mixed effects (MLME) linear regression analysis. Validity of BIS was demonstrated by resistance variables significantly decreasing with increasing net ionic fluid shift and increased TBSA (severity of injury) and calculated fluid volumes increasing with increasing net fluid shift and TBSA. BIS resistance also decreased with time as oedema reduced. For clinical use, a calculator was developed to adjust BIS variables when an Acticoat™ dressing is in situ, thus facilitating BIS variable change estimates in real time, with dressings intact.

**Conclusion:**

BIS may be used clinically to monitor fluid volume change in major acute burns.

**Electronic supplementary material:**

The online version of this article (10.1186/s41038-017-0105-9) contains supplementary material, which is available to authorized users.

## Background

Large fluid shifts and local and distant tissue swelling are features of burn injuries. Swelling hampers burn wound healing and the volume created is directly related to the size and depth of the burn [1]. Major burns greater than 15–20% total body surface area (TBSA) with a depth of partial to full thickness result in both a local and systemic inflammatory response [2, 3]. This can be a life-threatening scenario which requires formal fluid resuscitation. Acute burn fluid resuscitation is vital in decreasing patient morbidity and mortality in the first 24–48 h of injury but can contribute to already large amounts of oedema [4].

Despite the importance of fluid resuscitation in the early management of traumatic burn injuries, there is currently no single, simple, non-invasive and accurate outcome measure which can assist clinicians to titrate fluid volumes in acute burns or monitor the effect of treatments on swelling. Thus, the objective, timely adjustment of fluid resuscitation is challenging, particularly when patients are not supported by critical care and invasive monitoring. This research investigates the accuracy of bioimpedance spectroscopy (BIS) in monitoring whole-body fluid volume and oedema change in moderate to large acute burns.

There has been little advancement in the area of burn fluid resuscitation over the last 30 years [4] and in recent times, there has been a trend to over resuscitate patients [5, 6], necessitating a descriptor known as fluid creep. Excess fluid can contribute to burn wound progression, leading to complications such as peripheral and abdominal compartment syndromes, pulmonary oedema and peripheral tissue oedema. Any one or a combination of these will affect patient recovery and increase medical costs and is likely to increase patient length of stay [3, 7–10].

Fluid resuscitation formulas such as the Parkland and Brookes are used to instigate intravenous (IV) fluid rates but are guidelines only, and fluid must then be titrated according to particular endpoints of resuscitation [11–13]. The most commonly used outcome measure for fluid therapy is urine output, with the aim to maintain a rate of 30–50 ml per hour for an average-sized man while preserving haemodynamic properties such as oxygen saturation and blood pressure [5, 14]. There are other objective measures to guide volume titration however they are invasive and not without limitations [6, 14, 15].

BIS has historically been used in healthy populations to measure body composition. However, in the last 20 years, it has gained increasing popularity in clinical populations and is now commonly used to measure arm lymphoedema post breast surgery [16] and dry weight in haemodialysis patients [17, 18]. BIS has demonstrated sensitivity, high reliability (repeatability) of measures in a number of clinical areas [19]. The method has also been validated (determined credible) in both healthy and clinical populations against magnetic resonance imaging (MRI) and bromide and potassium dilution techniques, which are considered gold standard in the assessment of fluid compartment volumes and lean body mass (LBM) [20–23]. It can investigate the body’s physiological parameters such as extracellular fluid (ECF), intracellular fluid (ICF) and total body fluid (TBF). It achieves this by passing a small alternating current, over a number of frequencies (4–1000 kHz), through the tissues and fluid compartments of the body via electrodes on intact skin. It provides instantaneous measures of resistance (R) and reactance (capacitive resistance (Xc)). Resistance is the opposition to flow of an electric current, is reflective of the body’s water compartments and is inversely proportional to fluid volume and therefore oedema [24, 25]. Capacitance is the delay in the passage of current through the cell membranes and tissue interfaces [25]. The current flow is frequency (Hz) dependent and varies according to the composition of the body [26]. Resistances at zero (R_0_) and infinite frequencies (R_inf_) (considered ideal measurement frequencies) are estimated utilising the Cole-Cole plot embedded in the BIS software, due to the constraints of using a direct or very high frequency alternating current in humans [27]. The *R*_0_ and *R*_inf_ [25] are representative of ECF and TBF respectively. Resistance (*R*_i_) of the ICF is extrapolated using the other raw variable data. At low frequencies, the current can penetrate the ECF only, and at high frequencies, it passes through both the ECF and ICF measuring TBF.

The ability of BIS to quantify individual body fluid compartments, the ease of use and non-invasive nature has led to a small number of papers examining its use in the burn population. Miller et al. [28] and Zdolsek et al. [29] were able to determine the development of oedema post burn injury but each study lacked power and neither was able to provide statistical conclusions regarding the reliability of BIS in the burns populace. In 2009, Edgar et al. demonstrated whole-body bioimpedance spectroscopy was a reliable means of quantifying real-time oedema shifts in patients with burns less than 30% TBSA across numerous dressing conditions [30]. However, the study only had six participants with burns greater than 15% TBSA and was therefore inconclusive in this subset of patients. Further, each study utilised standard whole-body electrode positions only and it is unknown whether alternate electrode positions, for both whole-body and limb segmental BIS, are reliable in this particular population. Grisbrook et al. (2015) investigated whether alternate electrode configuration BIS measurements were interchangeable with standard electrode configurations in the healthy population but reliability was not determined [31]. In Edgar et al’s (2009) study [30], it was also apparent that the dressing condition affected the sensitivity of the BIS results. Bioimpedance measures were found to be less sensitive in older dressings (> 8 h old) than in an open wound or new dressing condition.

Dressing-type may pose a further challenge in the assessment of fluid shifts by BIS. Acticoat™ (Smith & Nephew) is an antimicrobial dressing, composed of nanocrystalline silver particles [32]. It is the standard dressing used in the first 48 h of burn care, and as indicated after, in the Burn Service of Western Australia (BSWA). Understanding that BIS measures the resistance of the body’s tissues and inter-compartmental fluid volumes by introducing a low amplitude electrical current into the body, it would not be unexpected that Acticoat™ may affect the BIS measures. Silver is a highly conductive material, and such dressings release ionic silver species and are applied in a wet condition. Both the silver ions and wet condition would therefore be expected to reduce the BIS resistance measured, thus potentially limiting the use of monitoring fluid shifts with BIS in acute burns patients.

To extend Edgar et al.’s (2009) [30] reliability study and on the premise that BIS can reliably quantify tissue fluid, it was hypothesised BIS would provide a method for real-time accurate measures of fluid shifts in the acute major burn. The study aimed to examine the reliability with respect to dressing condition and electrode position, investigate the influence of Acticoat™ on BIS variable outputs and determine the validity of whole-body BIS to assess net fluid shift in the presence of moderate to major burns, greater than 15% TBSA.

## Methods

### Participants

An observational longitudinal cohort study was conducted from December 2014 to February 2016. Patients were recruited into the study if they were over 18 years old and receiving formal fluid resuscitation and had a flame and/or scald burn, and the injury was less than 48 h old. The BSWA medical team instigates fluid resuscitation for partial to deep thickness burns greater than 15% TBSA (modified however based on each individuals clinical presentation and nutritional status at admission) and uses Ringer’s Lactate (crystalloid) solution with volumes initially determined by the modified Parkland’s formula. Fluid volumes were titrated to maintain an adequate urine output of 0.5–1.0 ml/kg/h for the first 36–48 h after burn injury. Participants were excluded from the research if they had hand and/or feet burns precluding placement of standard whole-body electrode placement and body mass index (BMI) ≤ 15 and ≥ 40 kg/m^2^ (manufacturer’s guidelines) and if they met Impedimed SFB7 (ImpediMed, Brisbane, Queensland, Australia) manufacturer’s contraindications which includes pregnant or breast-feeding patients, patients with surgical implants, cardiac pacemakers and/or are on electronic life support devices (ventilated patients).

Burn inpatients were recruited initially from the Burn Unit at Royal Perth Hospital (RPH) and then at Fiona Stanley Hospital (FSH) due to the transition of the adult care of the BSWA to the new FSH. There was no change to the study protocol or equipment used in the study.

### Equipment

**Fig. 1:**
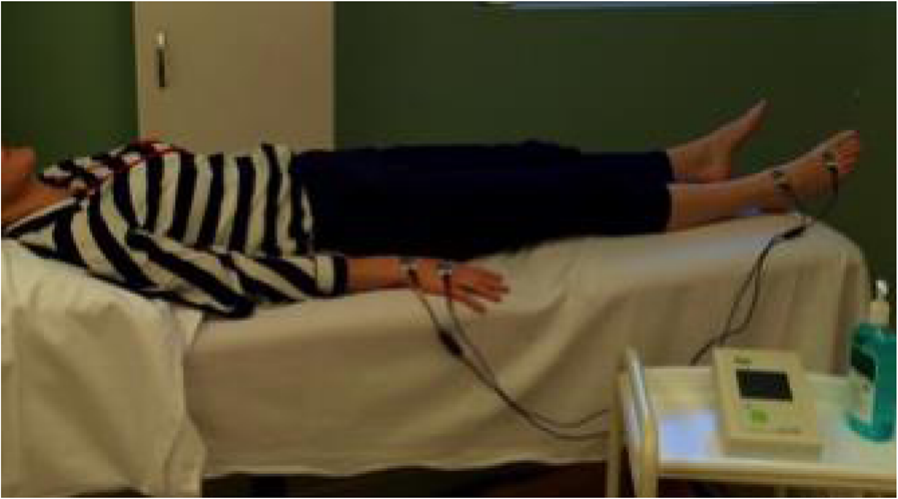
Bioimpedance spectroscopy (BIS): standard whole-body electrode positions

The ImpediMed SFB7 was used to collect whole-body and segmental BIS measures (Fig. 1). The calculated fluid volumes are stable when the subject’s BMI is > 15 kg/m^2^ (as per the manufacturer).

The BIS equipment measures both raw resistance variables and derived fluid distribution values such as whole-body ECF, ICF and TBF using the manufacturer’s algorithms. It achieves this by applying 256 discrete current frequencies (4–1000 Hz) through the body. ECF and ICF behave as resistive (R) components and R is inversely proportional to fluid volume [26, 33].

Diagnostic tab electrodes, Kendall CA610 (reference code 31447793, Covidien, Mansfield, MA, USA), were utilised.

### Procedures

Firstly, the patient’s weight and height was measured and input into the Impedimed instrument along with their age and gender. All BIS measures were taken using the manufacturer’s recommended and standardised positions with the patient lying supine and with the arms and legs abducted away from the body. BIS electrodes were placed over intact, cleaned skin (using alcohol swabs).

#### Electrode configurations

Standardised tetrapolar electrode placements (EP) were utilised [25, 34], and alternate electrode configurations were placed based on the theory of equi-potentials (see Cornish et al. [34] for further details of equipotential points) and were placed as per Grisbrook et al. [31]. Electrodes were placed on intact skin only. Participants with bilateral hand or foot injuries which precluded the application of standardised electrode placements were excluded. Bioimpedance measures were taken on the right side of the body unless precluded by wounds, then the left side was utilised. The location of their wounds determined whether all other electrode placements (segmental) could be used and measured.

**Fig. 2:**
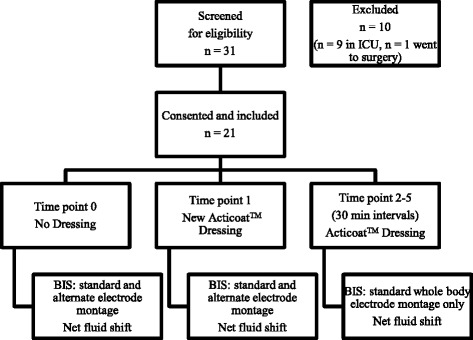
Consort diagram-flow diagram of data collection process. *ICU* Intensive care unit, *BIS*, Bioimpedance spectroscopy

BIS measures were taken in triplicate in an open wound (time point 0 (T0)) and in the new Acticoat™ dressing condition at five half-hour intervals (T1–T5) after the baseline measure, i.e. five measures in total (Fig. 2). The time between T0 and T1 was recorded, as this was unable to be standardised. Standard and alternate whole-body, upper limb segmental and lower limb segmental BIS measures were taken at T0–T1. Standard whole-body EP’s only were utilised at T2–T5 (Fig. 2). Burn wounds often prevent electrodes being applied in the standard position; therefore, alternative whole-body and limb segment electrode positions were utilised as able at T0–T1 and their reliability investigated. The data to determine the validity of alternate electrode placement has been analysed separately [35]. The segmental measures were included in the reliability analysis only. The effect of Acticoat™ on whole-body BIS results was determined from T0-T1 BIS readings. Electrodes remained in situ between triplicate measures where possible, unless prohibited by dressing changes or adhesive loss.

Net fluid shift was recorded between each time point (T1–T5), in conjunction with the BIS measures. Net fluid shift was calculated by subtracting urine output and other bodily fluid output recorded (e.g. emesis) from fluid intake (IV and oral fluids and food).

The researcher was blinded to all BIS measurements as only a file name was viewed and recorded, not the actual BIS values.

### Data analysis

Stata statistical software, release 14 (StataCorp LP 2014, College Station, TX), was utilised to analyse all results. Descriptive analyses were performed and were reported using the means and standard deviations (SD).

#### Reliability

A three-level nested mixed effects linear regression was performed to examine the reliability of the BIS triplicate measures, taking into account random effects of confounders of electrode position, time and dressing condition. The multilevel mixed effects (MLME) linear regression also explored whether there was a significant within-session difference between the triplicate measures for each of the BIS variables. Reliability is presented as the intra-class correlation coefficient (ICC) (acceptable, 0.75–0.89, excellent ≥ 0.9) [36], variance indicated by 95% confidence intervals (CI) and systematic bias between within session trial measures (*p* < 0.05 considered significant). All BIS triplicate measures were used in the analysis.

Analysis was completed using the MLME model as it can account for random effects from individuals and responses within individuals [37]. It is a robust method providing hierarchical analysis, adjusting for nested observations of measures for each individual and giving the most precise and least biased estimates of treatment effects. Prior to interpreting the results of the MLME, several assumptions were evaluated, confirming that each variable in the regression was approximately normally distributed.

#### Factors influencing BIS readings

The effect of dressing condition, %TBSA and initial TBF on the BIS whole-body variables only was determined by MLME linear regression. A separate model was performed for each BIS variable. The interaction between Acticoat™ and %TBSA and their influence on the BIS variables was also examined. The whole-body standard and alternate electrode placement BIS variable outputs were grouped together for use in the analysis for the effect of Acticoat™ and %TBSA. T0 (open wound) and T1 (new Acticoat™ dressing) were used only.

#### Validity

Validity was determined using a series of MLME linear regression models including the data with the Acticoat™ dressing condition only, and whole-body standard electrode placement (T1–T5) and alternate electrode placement (T1) only. The final model was produced by completing step-wise, backward elimination of predictor variables on each of the dependent BIS variables. The final model included %TBSA, time, net fluid shift and initial TBF volume. Initial TBF volume was derived from the mean of the TBF measured with an open wound using standard tetrapolar whole-body electrode placement as single-frequency bioimpedance analysis has been shown to measure TBF accurately in burns patients with no dressings [38]. This provided a baseline total body volume (L). A correlation matrix was performed to determine the relationship between initial TBF, weight and height and the skewness-kurtosis test demonstrated that they were each normally distributed.

Change scores or calculated difference of the BIS variables between time points (e.g. *R*_0_ at T1–*R*_0_ at T2) were not used in the validity analysis, as the calculation of a change score requires measurement of the outcome twice, and in practice, it is proposed that it is more efficient to use a (single) change from baseline measurement to derive outcomes. In addition by not analysing change (difference) data, the additive effect of the random errors is potentially reduced [39].

### Calculator

A calculator was developed to estimate the net fluid shift between consecutive BIS measures, when an Acticoat™ dressing is in place. Algorithms, for calculation of estimated fluid volumes were developed incorporating the significant and influential variables (on BIS variables) from the MLME models.

## Results

Twenty-one patients, 7 females and 14 males, were recruited post burn injury. One patient had an incomplete set of fluid recordings and two patients only had repeated measures completed four times in the new Acticoat™ dressing condition. The mean net fluid shift (SD) at each time point, separated by ~ 30 min for T1–T5, were as follows: T1 174.72 ml (533.18), T2 189.15 ml (164.23), T3 204.00 ml (135.37), T4 141.48 ml (253.25) and T5 123.20 ml (114.33). The average time between T0–T1 (SD) was 67 min (31). The mean TBF (SD) of patients on initial assessment was 46.06 L (9.71). Other patient data are presented in Table 1.

**Table 1:** Patient data (*n* = 21)

%TBSA	Age (years)	Recruitment post burn injury (h)	Height (cm)	Weight (kg)
24 (13) range 12–80	36.4 (13.5)	25 (11)	172.2	77.4 (16.3)

Values presented as means (SD) ± range

*TBSA* total body surface area

### Reliability

BIS triplicate measures were reliable within any electrode position, dressing condition and over time. Table 2 presents that BIS was a reliable measure in all circumstances, as confirmed by the ICC’s. There were no significant differences between the estimated means of within session triplicate trial measures for each of the BIS variables (i.e. no systematic bias) (Table 2). Final numbers included in each EP analysis were whole body standard (WBS) (*n* = 21), whole body alternate (WBA) (*n* = 18), upper limb standard (ULS) (*n* = 14), upper limb alternate (ULA) (*n* = 14), lower limb standard (LLS) (*n* = 15), lower limb alternate (LLA) (n = 14).

**Table 2:** BIS reliability

BIS variable	ICC (95% CI)	BIS trial number*	BIS measure coefficient (95% CI)	*p* value
*R*_0_	0.999 (0.999–0.999)	2	− 0.07 (− 0.68–0.54)	0.83
		3	− 0.06 (− 0.68–0.55)	0.84
*R*_i_	0.999 (0.998–0.999)	2	0.41 (− 1.90–2.71)	0.73
		3	2.06 (− 0.24–4.37)	0.80
*R*_inf_	0.9996(0.999–0.999)	2	0.01 (− 0.30–0.32)	0.94
		3	0.07 (− 0.24–0.38)	0.66
ECF	0.999 (0.998–0.999)	2	0.03 (− 0.17–0.22)	0.78
		3	0.12 (− 0.07–0.32)	0.22
ICF	0.997 (0.996–0.998)	2	− 0.12 (− 0.46–0.22)	0.49
		3	− 0.26 (− 0.61–0.08)	0.13
TBF	0.999 (0.999–0.999)	2	− 0.09 (− 0.38–0.20)	0.53
		3	− 0.14 (− 0.43–0.15)	0.33

*ICC* intraclass correlation coefficient, *R*_*0*_ resistance at zero frequency, *R*_*i*_ intracellular resistance, *R*_*inf*_ resistance at infinite frequency, *ECF* extracellular fluid, *ICF* intracellular fluid, *TBF* total body fluid, *BIS* bioimpedance spectroscopy, *CI* confidence intervals

*Each BIS measure coefficient is in reference to measure 1 of the triplicate measures

The means and CI for each of the BIS variables for the standard whole-body electrode placement and time point are presented in Table 3.

**Table 3:** BIS variable values for the standard whole-body electrode placement and time point

BIS variable at WBS	Time point					
	T0	T1	T2	T3	T4	T5
*R*_0_ (ohms)	498.77 (467.17–530.37)	351.94 (295.56–408.32)	366.70 (314.94–418.45)	371.18 (319.50–422.86)	371.76 (322.20–422.33)	401.01 (348.18–453.84)
*R*_i_ (ohms)	1412.47 (1225.51–1599.42)	715.75 (505.83–925.68)	715.51 (536.09894.93)	721.81 (546.31–897.31)	713.41 (541.38–885.44)	798.52 (611.02–986.02)
*R*_inf_ (ohms)	361.89 (337.57–386.20)	226.58 (183.50–269.67)	234.35 (195.19–273.52)	237.45 (198.23–276.67)	238.65 (200.24–277.06)	261.95 (220.50–303.40)
ECF (L)	20.76 (17.56–23.97)	34.77 (14.00–55.54)	32.50 (13.22–51.78)	31.93 (14.21–49.66)	31.50 (15.07–47.92)	24.84 (10.44–39.25)
ICF (L)	25.26 (21.62–28.91)	48.47 (27.74–69.21)	46.97 (27.11–66.83)	46.71 (27.15–66.27)	46.18 (27.20–65.16)	37.80 (21.38–54.23)
TBF (L)	46.03 (39.67–52.38)	83.16 (43.11–123.20)	79.48 (41.84–117.12)	78.53 (42.85–114.20)	77.65 (43.18–112.11)	62.65 (33.67–91.63)

Values presented as means (confidence intervals)

*BIS* bioimpedance spectroscopy, *WBS* standard whole-body electrode position, *R*_*0*_ resistance at zero frequency, *R*_*i*_ intracellular resistance, *R*_*inf*_ resistance at infinite frequency, *ECF* extracellular fluid, *ICF* intracellular fluid, *TBF* total body fluid, *T0* initial BIS measurement with no dressing, *T1* first BIS measure with new Acticoat™ dressing, *T2–T5* BIS measures taken at half hourly intervals

### Factors influencing BIS readings

The regression analysis demonstrated Acticoat™ had a significant effect on the raw variables *R*_i_ and *R*_inf_ (but not *R*_0_) and on all the calculated variables (ECF, ICF, TBF) in whole-body BIS (Table 4). The resistance variables reduced between 182.22 and 23.87 Ω for *R*_i_ and *R*_inf_, and the calculated volumes were increased by 31.00–67.23 L when an Acticoat™ dressing was in place, compared to the open wound condition.

**Table 4:** Predictor variable effects on whole-body BIS variables for determining the effect of Acticoat™

BIS variable	Covariate	Co-efficient	Confidence intervals		*p* value
			Lower	Upper	
*R*_0_ (ohms)	Acticoat™	− 17.42	− 39.35	4.52	0.12
	% TBSA	− 1.07	− 2.75	0.61	0.21
	Acticoat™#% TBSA	− 4.68	− 5.37	− 3.98	< 0.01*
	Initial TBF (L)	− 5.71	− 8.32	− 3.09	< 0.01*
*R*_i_ (ohms)	Acticoat™	− 182.22	− 265.27	− 99.16	< 0.01*
	% TBSA	6.50	− 3.45	16.46	0.20
	Acticoat™#% TBSA	− 17.98	− 20.61	− 15.36	< 0.01*
	Initial TBF (L)	− 32.52	− 48.16	− 16.87	< 0.01*
*R*_inf_ (ohms)	Acticoat™	− 23.87	− 38.57	− 9.17	< 0.01*
	% TBSA	− 0.01	− 1.33	1.32	0.99
	Acticoat™#% TBSA	− 3.96	− 4.42	− 3.49	< 0.01*
	Initial TBF (L)	− 5.30	− 7.37	− 3.23	< 0.01*
ECF (L)	Acticoat™	− 36.23	− 41.91	− 30.55	< 0.01*
	% TBSA	− 0.04	− 0.31	0.23	0.76
	Acticoat™#% TBSA	1.86	1.68	2.04	< 0.01*
	Initial TBF (L)	0.93	0.53	1.33	< 0.01*
ICF (L)	Acticoat™	− 31.00	− 36.07	− 25.92	< 0.01*
	% TBSA	− 0.15	− 0.36	0.07	0.18
	Acticoat™#% TBSA	2.01	1.85	2.17	< 0.01*
	Initial TBF (L)	1.08	0.77	1.40	< 0.01*
TBF (L)	Acticoat™	− 67.23	− 77.13	− 57.32	< 0.01*
	% TBSA	− 0.19	− 0.63	0.25	0.40
	Acticoat™#% TBSA	3.87	3.55	4.18	< 0.01*
	Initial TBF (L)	2.02	1.36	2.67	< 0.01*

Acticoat™ is in reference to an open wound

*R*_0_ resistance at zero frequency, *R*_i_ intracellular resistance, *R*_inf_ resistance at infinite frequency, *ECF* extracellular fluid, *ICF* intracellular fluid, *TBF* total body fluid, *TBSA* total body surface area, *BIS* bioimpedance spectroscopy

*p ≤ 0.05

#Interaction term

There was no evidence of an effect of TBSA on any of the BIS variables (Table 4). However, there was a statistically significant interaction (*p* < 0.01) between %TBSA and Acticoat™ for all BIS variables, raw and calculated. When an Acticoat™ dressing was in place and for every 1% increase in %TBSA *R*_0_ decreased by 4.68 Ω, *R*_i_ by 17.98 Ω and *R*_inf_ by 3.96 Ω. This results in a divergence away from the open wound R values as %TBSA increases. ECF, ICF and TBF volumes all increased with greater %TBSA when an Acticoat™ dressing was in place also resulting in divergence away from the open wound fluid volumes as %TBSA increased (Table 4).

As expected, there was a strong positive correlation between initial TBF and weight, with a correlation coefficient (r) of 0.83 (*p* < 0.01). There was also a moderate positive correlation between initial TBF and height, *r* = 0.67 (*p* < 0.01). Initial TBF was therefore included in the model, and height omitted, to reduce collinearity. Initial TBF was included in preference to BMI as it was determined to be a more robust indicator of a person’s size as the random error was reduced when compared to BMI (as it is one variable compared to two (height and weight)). Initial TBF is significantly associated with all BIS variables. For every 1 L increase in initial TBF, *R*_0_ decreased by 5.71 Ω (*p* < 0.01), *R*_i_ decreased by 32.52 Ω (*p* < 0.01) and *R*_inf_ decreased by 5.30 Ω (*p* < 0.01). All estimated fluid volumes increased (ECF 0.93 L, ICF 1.08 L, TBF 2.02 L) with every 1 L increase in initial TBF.

Algorithms were developed to correct for the effect of Acticoat™ for the BIS variables. They are as follows:

Corrected ECF = measured ECF with Acticoat dressing – (− 59.02 + (time since dressing applied × 1.38) + (initial measured ECF × 2.69))

Corrected ICF = measured ICF with Acticoat dressing – (− 79.26 + (time since dressing applied × − 0.0006) + (%TBSA × 1.85) + (initial measured ICF × 3.088918)).

### Validity

BIS resistance and fluid volume variables were analysed to determine BIS validity. The MLME linear regression univariate analysis, in the Acticoat™ dressing condition only, showed *R*_0,_ *R*_i_ and *R*_inf_ significantly changed with time (Table 5). Compared to T1 (new Acticoat™ dressing), for every minute increase in time, *R*_0_ decreased by 0.40 Ω (*p* < 0.01), *R*_i_ decreased 2.51 Ω (*p* < 0.01) and *R*_inf_ decreased 0.40 Ω (*p* < 0.01). The BIS-calculated fluid volumes ICF and TBF were also significantly associated with time, increasing by 60 and 20 ml for every minute increase in time (*p* < 0.01). ECF was not significantly associated with time.

**Table 5:** Univariate analysis of variable correlation on whole-body BIS measures

BIS variable	Covariate	Co-efficient	Confidence intervals		*p* value
			Lower	Upper	
*R*_0_ (ohms)	Time (minutes)	− 0.40	− 0.54	− 0.27	< 0.01*
	% TBSA	− 5.09	− 7.08	− 3.10	< 0.01*
	Net fluid shift (ml)	− 0.05	− 0.07	− 0.02	< 0.01*
	Initial TBF (L)	− 5.78	− 8.95	− 2.61	< 0.01*
*R*_i_ (ohms)	Time (minutes)	− 2.51	− 3.09	− 1.92	< 0.01*
	% TBSA	− 8.85	− 16.98	− 0.74	0.03*
	Net fluid shift (ml)	− 0.25	− 0.36	− 0.15	< 0.01*
	Initial TBF (L)	− 28.79	− 41.74	− 15.84	< 0.01*
*R*_inf_ (ohms)	Time (minutes)	− 0.40	− 0.51	− 0.28	< 0.01*
	% TBSA	− 3.25	− 4.69	− 1.81	< 0.01*
	Net fluid shift (ml)	− 0.05	− 0.07	− 0.03	< 0.01*
	Initial TBF (L)	− 5.38	− 7.68	− 3.07	< 0.01*
ECF (L)	Time (minutes)	0.02	− 0.01	0.05	0.15
	% TBSA	1.40	0.99	1.80	< 0.01*
	Net fluid shift (ml)	0.01	− 0.001	0.01	0.09
	Initial TBF (L)	1.20	0.56	1.85	< 0.01*
ICF (L)	Time (minutes)	0.06	0.03	0.10	< 0.01*
	% TBSA	1.52	1.17	1.88	< 0.01*
	Net fluid shift (ml)	0.01	0.01	0.02	< 0.01*
	Initial TBF (L)	1.56	0.99	2.13	< 0.01*
TBF (L)	Time (minutes)	0.08	0.02	0.14	< 0.01*
	% TBSA	2.92	2.18	3.65	< 0.01*
	Net fluid shift (ml)	0.02	0.01	0.03	< 0.01*
	Initial TBF (L)	2.77	1.59	3.94	< 0.01*

*R*_*0*_ resistance at zero frequency, *R*_*i*_ intracellular resistance, *R*_*inf*_ resistance at infinite frequency, *ECF* extracellular fluid, *ICF* intracellular fluid, *TBF* total body fluid, *TBSA* total body surface area, *BIS* bioimpedance spectroscopy

*p ≤ 0.05

**Fig. 3:**
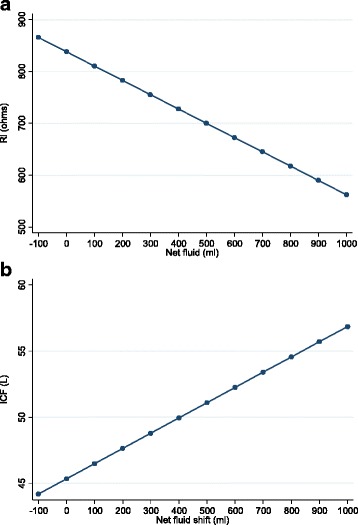
**a**, **b** Predicted margin plots of BIS variable (*R*_i_, ICF) and net fluid shift relationship. *The predicted margin plots of *R*_0_ and *R*_inf_ have a similar linear relationship to net fluid shift as *R*_i._ ECF and TBF have a similar linear relationship to net fluid shift as ICF. *R*_0_, Resistance at zero frequency; *R*_i_, Intercellular resistance; *R*_inf_, Resistance at infinite frequency; *ECF*, Extracelluar fluid; *ICF*, intracellular fluid; *TBF*, total body fluid

The regression analyses demonstrated all resistance values significantly decreased with increasing net fluid volume in a linear relationship (Table 5, Fig. 3a). Net fluid volume was significantly associated with ICF and TBF BIS fluid volume change, increasing with increasing net fluid shift (Fig. 3b). All BIS variables were significantly associated with % TBSA. For every 1% increase in TBSA, *R*_0_ decreased by 5.09 Ω, *R*_i_ decreased 8.85 Ω and *R*_inf_ decreased 3.25 Ω. Fluid volumes increased between 1.20–2.77 L with every 1% increase in TBSA (*p* < 0.01) (Table 5).

Two individuals who had large negative fluid shifts > 850 ml across a single time point were removed from the analysis after the stepwise analysis found that they significantly altered the results of the final model. Leaving these patients in the analysis would have resulted in a non-homogenous sample. It appears a large loss of fluid volume compromises the interpretation of BIS measures. Both patients suffered loss of large volumes of ionic fluid due to emesis which likely altered the measured BIS resistance [27].

When a patient’s initial TBF increased by 1 L, *R*_0_ decreased by 5.78 Ω (*p* < 0.01), *R*_i_ decreased 28.79 Ω (*p* < 0.01) and *R*_inf_ decreased 5.31 Ω (*p* < 0.01).

### Calculator

A calculator was developed to estimate the net fluid shift between consecutive BIS measures, accounting for dressing condition, %TBSA and time since dressing (Additional file 1). The significant and influential variables from the MLME models (Table 5) were incorporated into the newly developed algorithms (for calculation of fluid volumes), which were then embedded in an excel calculator to allow clinicians access to them. The variables required for input into the calculator by the clinician include dressing condition, %TBSA, time since application of Acticoat™ dressing (minutes) and the measured BIS variables. The calculator does not require the clinician to monitor or include net fluid shift, namely urine output and fluid input.

The validity analysis utilised the measured BIS fluid volumes and did not correct for the Acticoat™ effect, as it was not considered necessary for this preliminary study.

## Discussion

The principal novel finding of this study show BIS was a reliable method for monitoring fluid change in moderate to large burn patients. Bioimpedance resistance measures can be interpreted in the presence of Acticoat™ to monitor changes in fluid volume over time, if corrected for using the provided calculator. Thus, the study also established BIS as a valid indicator of fluid change over time during burns resuscitation while Acticoat™ dressings are in situ. BIS at the bedside has the potential to improve fluid management in an acute major burn by providing real-time measures of fluid shifts thus reducing the risk of over resuscitation and associated adverse outcomes.

### Reliability

The results of the study demonstrate BIS produces reliable raw and predicted measures in patients with > 12% TBSA burns, regardless of dressing condition (open wound or Acticoat™) and electrode placement (Table 5). This data suggests BIS is a reliable method for assessing oedema change over time in moderate to large area burns. This concurs with and adds to the findings of Edgar et al.’s (2009) study which found BIS reliability applicable to burns with < 30% TBSA across different dressing conditions [30].

### Factors influencing BIS readings

Whole-body bioimpedance-calculated fluid volumes were grossly and significantly overestimated, and resistance of the ICF and TBF was underestimated when an Acticoat™ dressing was in place. The under or overestimation of BIS variables increased with increasing TBSA. Grisbrook et al. [40] and Kenworthy et al. [35] also found that the effect of silver dressings on BIS variable measures increased with increasing size of the burn.

BMI is also well known to be associated with BIS variable output as larger people have a greater amount of body fluid [41]. This has been demonstrated in the present results where a larger initial TBF (indication of the bulk of the person and collinear with BMI) significantly decreased BIS resistance and therefore increased calculated fluid volumes.

It can be concluded that BIS was appropriate for use in a moderate to large burns population when an Acticoat™ dressing was in place only with adjustment, as resistance measures and fluid volumes are significantly under and overestimated with significantly different values to those in an open wound. The SFB7 impedimed-embedded algorithms are not appropriate for use in burns with Acticoat™ in situ. This is consistent with the findings of Grisbrook et al. [40] though the burns population sample in that study did differ from those recruited in this study sample with respect to %TBSA (range 5.5–28.5% compared to our 12–80%) and fluid resuscitation requirements. Therefore, to monitor fluid shifts, it is recommended that the resistance and fluid volume variables measured when an Acticoat™ dressing, in situ, be corrected using the provided calculator.

### Validity

The present results show that BIS is a valid indicator of fluid volume change over time in moderate to large burn resuscitation with TBSA, time, net fluid shift and initial TBF all significantly associated with BIS resistance and calculated fluid volumes. For clinically interpretable results, the measured BIS variables need to be adjusted using the provided calculator if Acticoat™ is in place.

Time was significantly associated with resistance variables, with an increase in time decreasing all estimated resistances and increasing ICF and TBF volumes. This may be explained by a combination of factors including the time since dressing application, the effect of Acticoat™ and the amount of fluid resuscitation administered. Firstly, over time the Acticoat™ dressing deposits more silver ions into the wound, therefore decreasing the raw resistance values and in turn increasing the ‘equivalent’ fluid volumes as calculated by BIS-embedded algorithms [42]. Secondly, the total mean volume of fluid resuscitation over time increased, thus increasing all inter-compartmental fluid volumes and consequentially decreasing the associated estimated resistance values. Although ECF was not associated with time, the *p* value (0.15) is arguably low enough to accept that a clinical relationship may exist despite a small sample. In contrast, the embedded algorithm of analysis may explain why ECF is not associated with time in this population (each algorithm has different constants for estimating the individual fluid compartments [43]). However, *R*_0_, the equivalent resistance of ECF significantly changed with time, suggesting fluid volume change in the extracellular compartment is associated with time.

It is known that BIS resistance is inversely proportional to fluid volume [22, 24]. The results of this study support this. Bioimpedance variables and net fluid shift were found to have a negative inverse linear relationship with resistance and as expected, calculated fluid volumes a positive linear relationship (Fig. 3) provided that the net fluid shift (at each half hour measure) was greater than 100 ml. There were two patients who had a large (> 850 ml) negative fluid shift, both noted to have emesis during the single measurement period, and thus, these data were excluded from the analysis, as they were assumed to have an altered, uncorrected physiological (ionic) state at the time of measurement and thus, significantly differed from others in the sample. It appears that a large loss of fluid consequentially affects the following repeated BIS measures (within at least the following 2 h). It is proposed that not only was the volume change a contributor to the difficulty in interpretation of the BIS measures but also the loss of electrolytes from the gut following emesis. The emesis could have altered the whole-body fluid ionic state for a short period until it was corrected by the body systems. Bioimpedance resistance is inversely proportional to fluid volume and electrolyte concentration. Therefore, significant changes in the ionic status of the fluid or tissues measured will alter the BIS raw variables and render the machine-embedded algorithms for calculated volumes invalid. Clinicians are advised not to use BIS measures in the period after an episode of emesis [27]. Further, the results suggest the BIS measure is only sensitive to fluid losses ≤ 100 ml per half hour in the burns resuscitation period. The sensitivity of the BIS measure for fluid losses greater than 100 ml and less than 850 ml cannot be predicted as the patient cohort did not experience losses in this range.

### Calculator

On the basis of the results a calculator was developed to improve the clinical utility of BIS in burns resuscitation patients at the bedside. It adjusts for the Acticoat™ effect and provides an estimated change in BIS resistance and fluid volumes between consecutive BIS measurements, hence allowing fluids to be titrated accordingly. It has been established however that BIS is reliable and valid in the open wound condition. Therefore, BIS can be utilised without variable adjustment when no dressings are in place.

### Clinical practice recommendations

Optimum fluid resuscitation requires maintenance of the intracellular volume with minimal expansion (extravasation) of the extracellular volume. The results of this study indicate that using the relationship or pattern between *R*_0_ or ECF and *R*_i_ or ICF is a non-invasive, interpretable method of monitoring or titrating fluid resuscitation. A stabilised *R*_i_ or ICF volume, over time, equal to or greater than the normal range (ICF 22.9–25 L) [24] represents a fluid resuscitation target. Fluid volumes should then be titrated to maintain *R*_0_ or ECF at a steady state whilst continuing to preserve *R*_i_ or ICF at the target volume. Ideally ECF volumes would be maintained as close to normal (or the average for a healthy person) as possible (13.2–15.3 L). However due to the body’s systemic “leaky vessel” inflammatory response to a major burn injury, with extravasation of fluid into the extracellular space, volumes within 5–10% of these norms would be a suggested acceptable target range [44, 45]. In postoperative surgical patients, fluid overload has been defined as > 15% of preoperative fluid volume [44] and in haemodialysis patients reaching ECF volumes within one to two litres of normal values is deemed acceptable [46]. An example of how to titrate fluids: If *R*_i_ or ICF is stable and the change values of *R*_0_ or ECF continue to increase, the fluid administered is adding to the extracellular compartment (swelling) rather than preferentially maintaining the intracellular compartment. Infused fluid volumes therefore need to be reduced if *R*_i_ (ICF) is stable and *R*_0_ (ECF) is trending upward. However, in a recent study, intracellular volume actually decreased (~ 0.8 L over 70 min) upon rapid infusion of intravenous fluid (~ 2 L in ~ 60 min) into healthy male volunteers [47]. It was suggested that the infusion of fluid was responsible for the increase in ECF. The fluid administered in this study was < 500 ml/h; therefore, it is difficult to conclude whether this may have the same effect. It however does suggest potentially accepting an ICF volume of ~ 1 L less than the average volumes when considering titrating fluid as above. For greater sensitivity to change, at this time, this study suggests that it is more advantageous to use the change in BIS raw resistance values (adjusted in the presence of Acticoat™) rather than the calculated volumes as it removes the need for specific predictive equations and eliminates the need for height and weight measures [48]. There are a growing number of studies suggesting that raw BIS variables may be more useful in predicting clinical outcomes [49, 50]. BIS raw variables may also be able to indicate changes associated with cell membrane damage and cell wall integrity [50].

Further work is required to increase the confidence and promote greater utility of this sensitive measure over standard haemodynamic monitoring. In contrast, urine output, a ‘quasi’ measure of fluid shifts and whole-body perfusion [8] has been suggested to lag behind the actual events of hypoperfusion by up to 2 h [51, 52]. Bioimpedance also removes the need to rely heavily on initial fluid volume calculations such as the Parkland or Brooke’s. This proves to be highly useful out in the field with paramedics and in isolated country hospitals where clinician’s burns experience may be limited and where Western Australia’s vastness means it is not uncommon for people to travel greater than 8 h to be admitted to a tertiary hospital.

### Future research

Additional research is warranted in evaluating the effect of other silver and non-silver dressings such as sulfadiazine and hydrocolloids, in moderate to large burns to increase the utility of BIS across burns services.

Further, consideration may need to be given of the type of resuscitation fluid (e.g. crystalloids versus colloids) in future studies as BIS electrical conductivity is affected by electrolyte concentration. This may therefore influence BIS variable measurements. Electrical and chemical burn injuries may also influence or change the ionic state of the tissue. Thus, future research should include these modes of injury.

Ideally, BIS would be able to be used on burns patients on life support or mechanical ventilation however further study needs to be done to determine whether electronic equipment interferes with the BIS instrument. Several studies have been conducted in intensive care units however they did not stipulate whether ventilated patients were included [53, 54].

## Conclusions

In moderate to large burn patients, BIS is a reliable and valid method of oedema change. The Acticoat™ dressings significantly alter the BIS raw outputs. To allow clinical interpretation of BIS, measures must be adjusted for silver dressings.

## Additional files

Additional file 1: Acticoat calculator for oedema—excel spreadsheet. (XLSX 13 kb)
